# Association between clopidogrel preloading time and post-procedural troponin elevation in patients with stable angina undergoing elective percutaneous coronary intervention: a retrospective cohort study

**DOI:** 10.12701/jyms.2026.43.34

**Published:** 2026-05-18

**Authors:** Sungho Jo, Jeong Tae Byoun, Donghyeon Joo, Jae Young Cho, Kyeong Ho Yun

**Affiliations:** Department of Cardiovascular Medicine, Regional Cardiocerebrovascular Center, Wonkwang University Hospital, Iksan, Korea

**Keywords:** Angina pectoris, Clopidogrel, Preoperative care, Time factors, Troponin

## Abstract

**Background:**

Clopidogrel requires several hours to achieve adequate platelet inhibition. We investigated the association of clopidogrel preloading time with 30-day clinical outcomes and post-procedural troponin elevation in patients with stable angina undergoing elective percutaneous coronary intervention (PCI).

**Methods:**

This single-center retrospective cohort study included 1,020 patients with stable angina (clopidogrel-naive) who received 300 mg clopidogrel preloading within 24 hours before elective PCI between 2012 and 2020. Patients were categorized according to clopidogrel preloading-to-balloon time ≤6 hours or >6 hours. The primary endpoint was 30-day major adverse cardiovascular events (MACE), defined as a composite of all-cause death, myocardial infarction, stroke, and any revascularization. Secondary endpoints included serial troponin T changes and troponin T elevation ≥5×, ≥25×, and ≥70× the upper reference limit. Stabilized inverse probability of treatment weighting (IPTW) was used.

**Results:**

Thirty-day MACE occurred in five patients (0.49%) and did not differ between the ≤6-hour and >6-hour groups after IPTW (0.5% vs. 0.4%, *p*=0.754). Post-procedural troponin T levels at 6, 24, and 48 hours were higher in the ≤6-hour group. Patients with shorter preloading-to-balloon times showed higher peak troponin T levels, with the greatest difference at the ≤1-hour cutoff (geometric mean ratio, 2.12; 95% confidence interval, 1.53–2.95; *p*<0.001) and progressive attenuation at longer cutoffs. Troponin T elevation ≥5× and ≥25× was more frequent in the ≤6-hour group, whereas ≥70× elevation did not differ.

**Conclusion:**

Clopidogrel preloading ≤6 hours before elective PCI was not associated with increased 30-day MACE but was associated with lower-threshold post-procedural troponin T elevation.

## Introduction

Platelet inhibition using aspirin and P2Y12 inhibitors is a standard of care for patients undergoing percutaneous coronary intervention (PCI) with drug-eluting stents. To maximize the antiplatelet effect of P2Y12 inhibitors, theoretically, pretreatment or use of potent P2Y12 inhibitors is essential, especially in patients with a high thrombotic risk, such as myocardial infarction. P2Y12 inhibitor pretreatment refers to the administration of a P2Y12 inhibitor before coronary angiography, while preloading is often used synonymously but specifically refers to the upstream administration of a P2Y12 inhibitor loading dose [1]. However, studies using pretreatment with the potent P2Y12 inhibitors prasugrel and ticagrelor did not show a clinical benefit of pretreatment over treatment in the catheterization laboratory in patients with myocardial infarction [2,3]. Therefore, the current guidelines do not recommend routine pretreatment with P2Y12 inhibitors in patients with acute coronary syndrome, particularly when the coronary anatomy is unknown [4].

Clopidogrel is a prodrug requiring two-step bioactivation, and clinical responses are variable due to its complex pharmacokinetics and the influence of genetic polymorphisms [5]. Despite these limitations, clopidogrel is the most widely used drug for the treatment of patients with angina. The effects of clopidogrel preloading in patients with angina were first studied in the Clopidogrel for the Reduction of Events During Observation (CREDO) trial [6]. The investigators found that, whereas 300 mg of clopidogrel preloading for <6 hours was not associated with a reduction in composite major adverse cardiovascular events (MACE), preloading for >15 hours could significantly reduce adverse events [7]. However, other prospective or observational studies have shown the opposite results, even though they used a higher dose and longer duration of clopidogrel pretreatment [8,9].

In contemporary practice, same-day coronary angiography and PCI are frequently performed, which may limit the feasible duration of P2Y12 inhibitor treatment before PCI. Clopidogrel requires metabolic activation and has a relatively delayed onset of platelet inhibition; therefore, a loading dose of 300 to 600 mg is commonly administered before PCI in patients who are clopidogrel-naive. However, whether the timing of clopidogrel loading before PCI influences clinical outcomes or post-procedural biomarker release remains unclear. In this study, we evaluated the association of clopidogrel preloading time with 30-day clinical events and serial post-procedural troponin T elevation in patients with stable angina and no prior exposure to clopidogrel who underwent elective PCI after 300 mg clopidogrel preloading within 24 hours before the procedure. Patients were categorized using a 6-hour cutoff for clopidogrel preloading-to-balloon time, based on the timing analysis of the CREDO trial.

## Methods

**Ethics statement:** This study protocol was reviewed and approved by the Institutional Review Board (IRB) including the Ethics Committee of Wonkwang University Hospital (IRB No: 2024-05-020). Informed consent was waived by the IRB due to its retrospective and observational nature and the absence of any patient-identifying information.

### 1. Study population

**Fig. 1.**
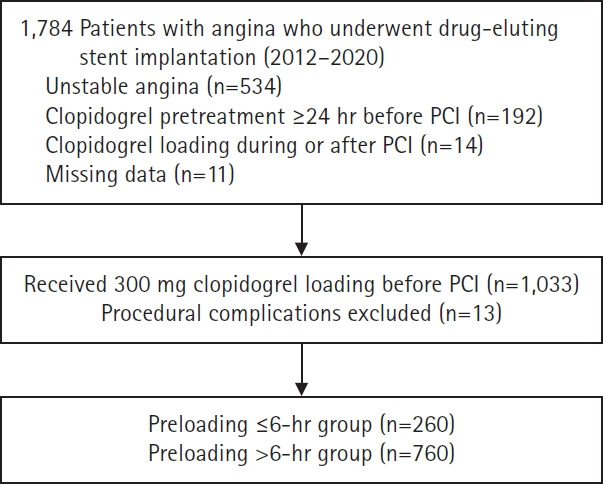
Patient selection flow diagram. PCI, percutaneous coronary intervention.

We retrospectively analyzed a single-center cohort of patients with angina who underwent elective PCI between 2012 and 2020. Patients were eligible if they were aged ≥18 years, underwent successful implantation of a second-generation drug-eluting stent, and had an expected life expectancy of >1 year. Patients were excluded if they had unstable angina, received preloading with a P2Y12 inhibitor other than clopidogrel, received clopidogrel pretreatment for >24 hours before PCI, received clopidogrel loading in the catheterization laboratory or after PCI, or had missing data. To minimize the confounding effects of procedure-related myocardial injury, patients with major procedural complications, including no-reflow phenomenon, major coronary dissection, side branch occlusion, or distal embolization, were also excluded. The patient selection process is illustrated in Fig. 1. The final study population consisted of 1,020 patients with stable angina who received 300 mg clopidogrel preloading within 24 hours before elective PCI. Data on baseline clinical characteristics, PCI-related variables; laboratory findings at baseline and 6, 24, and 48 hours after PCI; and 30-day clinical outcomes were collected retrospectively. PCI was performed according to current clinical practice at the physician’s discretion using a second-generation drug-eluting stent. In all patients, 300 mg aspirin and 300 mg clopidogrel were administered before the procedure, and the preloading-to-balloon time was recorded. After PCI, aspirin (100 mg/day) and clopidogrel (75 mg/day) were prescribed to all patients. Creatine kinase MB fraction (CK-MB) and troponin T levels were measured at admission and 6 hours, 24 hours, and 48 hours after PCI. The upper reference limit (URL) of our laboratory was 0.014 ng/mL, and the lower detection limit was 0.003 ng/mL. If the troponin T level was below the detection range, we considered it to be 0.003 ng/mL. Additional samples were obtained if a patient showed signs or symptoms of myocardial ischemia.

### 2. Endpoints

The primary endpoint was 30-day MACE, defined as a composite of all-cause death, myocardial infarction, stroke, and any revascularization including target vessel revascularization and non-target vessel revascularization.

The secondary endpoints focused on post-procedural troponin T release. Serial troponin T levels were measured at baseline and 6, 24, and 48 hours after PCI and were compared between patients with a clopidogrel preloading-to-balloon time of ≤6 hours and those with a clopidogrel preloading-to-balloon time of >6 hours. Peak troponin T levels were further compared using cumulative preloading-to-balloon time cutoffs of 1, 2, 4, 6, 8, and 12 hours. Biomarker-defined periprocedural myocardial injury was evaluated using post-procedural troponin T elevation thresholds of ≥5×, ≥25×, and ≥70× the URL, based on previously reported thresholds [10-13]. These troponin-based endpoints were considered biomarker outcomes and were analyzed separately from clinical myocardial infarction. Bleeding complications were also assessed based on the Bleeding Academic Research Consortium (BARC) definition, specifically by examining BARC type 3 or 5 bleeding events [14].

### 3. Statistical analyses

Continuous variables are presented as mean±standard deviation or medians with interquartile ranges, as appropriate, and categorical variables are presented as numbers and percentages. Between-group comparisons were performed using the independent *t*-test or Mann-Whitney U-test for continuous variables and the chi-square test or Fisher exact test for categorical variables.

Crude odds ratios (ORs) with 95% confidence intervals (CIs) were calculated for the clinical and biomarker outcomes. To account for baseline differences between patients with clopidogrel preloading times of ≤6 hours and >6 hours, stabilized inverse probability of treatment weighting (IPTW) was performed using propensity scores estimated from a logistic regression model. The propensity score model included age, diabetes mellitus, previous myocardial infarction, multivessel disease, hemoglobin, estimated glomerular filtration rate (eGFR), baseline troponin T, low-density lipoprotein cholesterol, maximal balloon pressure, clopidogrel, and discharge medications. The covariate balance before and after weighting was assessed using standardized mean differences, with an absolute standardized mean difference <0.1 considered an acceptable balance.

Serial changes in troponin T levels were analyzed using a linear mixed-effects model with a random intercept for patients after log transformation of troponin T values. Because peak troponin T values were right-skewed, log-transformed values were used for between-group comparisons, and the results are presented as geometric mean ratios with 95% CIs. Multivariable logistic regression analysis was performed to identify independent predictors of biomarker-defined periprocedural myocardial injury. All *p*-values are two-sided, and statistical significance was defined as a *p*-value <0.05. Statistical analyses were performed using R version 4.5.2 (R Foundation for Statistical Computing, Vienna, Austria).

## Results

**Fig. 2.**
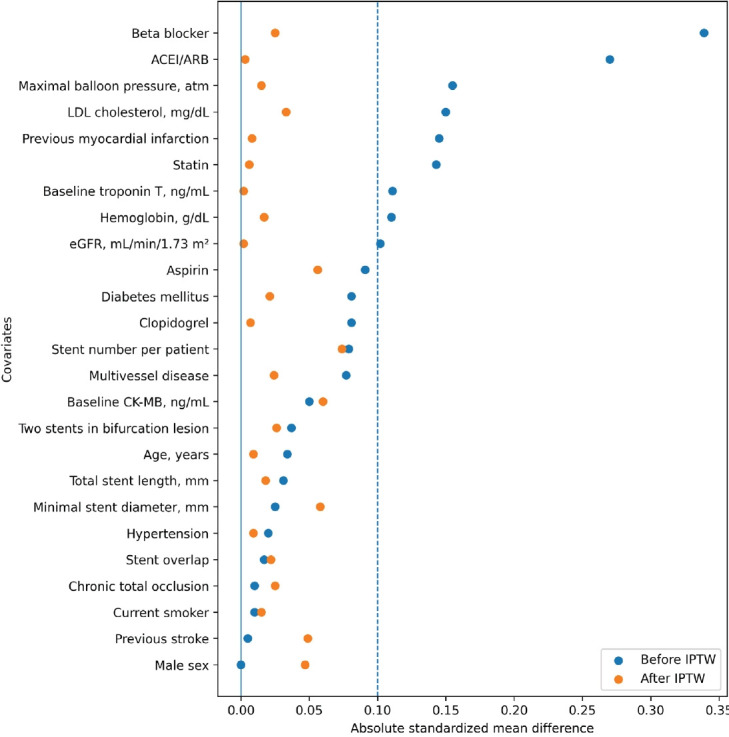
Covariate balance before and after inverse probability of treatment weighting (IPTW). Covariate balance is assessed using absolute standardized mean differences before and after IPTW. The dashed vertical line indicates an absolute standardized mean difference of 0.10. ACEI, angiotensin-converting enzyme inhibitor; ARB, angiotensin receptor blocker; LDL, low-density lipoprotein; eGFR, estimated glomerular filtration rate; CK-MB, creatine kinase MB fraction.

The baseline characteristics according to clopidogrel preloading time are presented in Table 1. Before IPTW, several clinical and treatment-related variables were imbalanced between the two groups. After stabilized IPTW, the covariate balance was substantially improved, with absolute standardized mean differences below 0.1 for most variables. The covariate balance before and after IPTW is shown in Fig. 2.

The primary endpoint, 30-day MACE, occurred in five patients (0.49%). Overall, 30-day clinical events were infrequent and showed no significant differences according to the 6-hour clopidogrel preloading-to-balloon time cutoff (Table 2). In the crude analysis, MACE occurred in two patients (0.8%) in the ≤6-hour group and in three patients (0.4%) in the >6-hour group (OR, 1.96; 95% CI, 0.33–11.77; *p*=0.606). After stabilized IPTW, the weighted incidence of MACE remained similar between the groups (0.5% vs. 0.4%; OR, 1.40; 95% CI, 0.17–11.38; *p*=0.754). The incidence of BARC type 3 or 5 bleeding was also not significantly different between the groups.

**Fig. 3.**
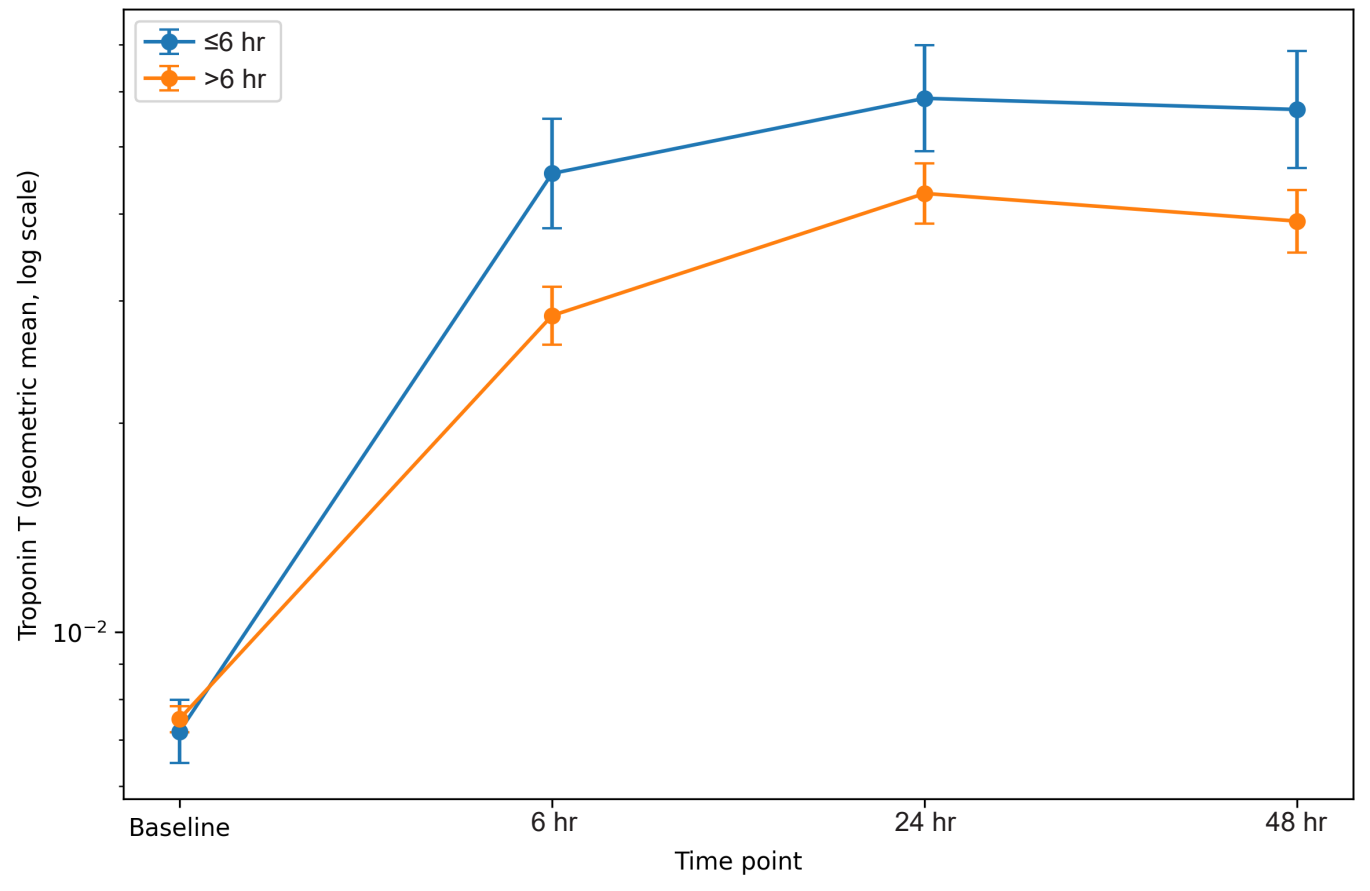
Serial changes in troponin T according to preloading-to-balloon time. Troponin T levels at baseline and at 6, 24, and 48 hours after percutaneous coronary intervention are shown according to clopidogrel preloading-to-balloon time ≤6 hours or >6 hours. Values are presented as geometric means with 95% confidence intervals.

Serial changes in troponin T levels according to the 6-hour clopidogrel preloading-to-balloon time cutoff are shown in Table 3 and Fig. 3. The baseline troponin T levels were similar between the two groups (geometric mean, 0.0075 vs. 0.0076 ng/mL; *p*=0.847). However, post-procedural troponin T levels were significantly higher in the ≤6-hour group at 6 hours (0.0462 vs. 0.0290 ng/mL; *p*<0.001), 24 hours (0.0592 vs. 0.0433 ng/mL; *p*<0.001), and 48 hours (0.0572 vs. 0.0396 ng/mL; *p*<0.001). In the linear mixed-effects model using log-transformed troponin T values, there was a significant group-by-time interaction (likelihood ratio, χ²=40.68; df=3; *p*<0.001), indicating that the temporal pattern of troponin T change differed according to clopidogrel preloading time.

**Fig. 4.**
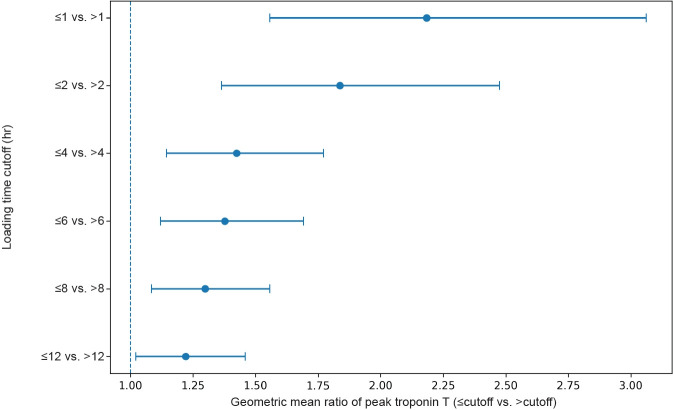
Geometric mean ratios of peak troponin T according to cumulative clopidogrel preloading-time cutoffs. Geometric mean ratios compare patients with clopidogrel preloading-to-balloon time at or below each cutoff with those above the cutoff. Horizontal bars indicate 95% confidence intervals.

Peak troponin T levels according to cumulative clopidogrel preloading-to-balloon time cutoffs are shown in Table 4 and Fig. 4. The geometric mean ratio for peak troponin T was 2.12 (95% CI, 1.53–2.95; *p*<0.001) for ≤1 hour versus >1 hour and 1.80 (95% CI, 1.35–2.40; *p*<0.001) for ≤2 hours versus >2 hours. The ratio decreased progressively with longer cumulative cutoffs: 1.40 (95% CI, 1.13–1.73; *p*=0.002) at ≤4 hours, 1.36 (95% CI, 1.11–1.66; *p*=0.003) at ≤6 hours, 1.28 (95% CI, 1.07–1.52; *p*=0.006) at ≤8 hours, and 1.21 (95% CI, 1.02–1.43; *p*=0.033) at ≤12 hours.

Biomarker-defined periprocedural myocardial injury according to the 6-hour clopidogrel preloading-to-balloon time cutoff is shown in Table 5. In the crude analysis, a troponin T elevation of ≥5× URL occurred more frequently in the ≤6-hour group than in the >6-hour group (43.1% vs. 33.6%; OR, 1.50; 95% CI, 1.12–2.00; *p*=0.006). This difference remained consistent after IPTW adjustment (43.6% vs. 33.7%; OR, 1.52; 95% CI, 1.13–2.04; *p*=0.006). A troponin T elevation of ≥25× URL was also more frequent in the ≤6-hour group in both the crude analysis (16.5% vs. 8.7%; OR, 2.08; 95% CI, 1.38–3.15; *p*<0.001) and the IPTW analysis (15.4% vs. 8.3%; OR, 2.03; 95% CI, 1.31–3.14; *p*=0.002). In contrast, a troponin T elevation of ≥70× URL did not differ between the groups in either the crude or IPTW analysis.

The multivariable logistic regression analysis for biomarker-defined periprocedural myocardial injury is presented in Table 6. Clopidogrel preloading ≤6 hours was independently associated with troponin T elevation of ≥5× URL (OR, 1.50; 95% CI, 1.10–2.05; *p*=0.010) and ≥25× URL (OR, 2.15; 95% CI, 1.38–3.34; *p*<0.001), but not with troponin T elevation of ≥70× URL (OR, 0.83; 95% CI, 0.36–1.90; *p*=0.658). Multivessel disease was associated with troponin T elevation of ≥5× URL and ≥25× URL, whereas eGFR <60 mL/min/1.73 m² was consistently associated with all biomarker-defined injury thresholds.

## Discussion

The main findings of this study are as follows. First, clopidogrel preloading ≤6 hours before balloon inflation was not associated with increased 30-day MACE, stent thrombosis, major bleeding, or net events in either the crude or IPTW analysis. Second, despite the absence of differences in short-term clinical outcomes, patients with clopidogrel preloading ≤6 hours showed greater serial increases in troponin T after PCI than those with longer preloading times. Third, clopidogrel preloading ≤6 hours was independently associated with lower-threshold biomarker-defined periprocedural myocardial injury, including troponin T elevations of ≥5× and ≥25× URL, but not with a severe troponin T elevation of ≥70× URL. These findings indicate a biomarker-level association between shorter clopidogrel preloading times and post-procedural troponin T release but do not establish an increase in clinically meaningful adverse events.

Oral P2Y12 inhibitors require time to achieve sufficient platelet inhibition. The CREDO trial suggested a time-dependent relationship between clopidogrel preloading duration and adverse cardiovascular events: preloading for <6 hours was not associated with benefit, whereas preloading for ≥6 hours showed a trend toward risk reduction, with a stronger effect after ≥15 hours [6,7]. Based on this prior timing analysis, we selected 6 hours as the primary cutoff for clopidogrel preloading-to-balloon time. This cutoff value is also biologically plausible. Clopidogrel requires intestinal absorption and hepatic activation, and a 300 mg loading dose achieves maximal platelet inhibition after approximately 4 to 5 hours, whereas a 600 mg loading dose has a faster onset of action [15,16]. In the present study, clopidogrel preloading ≤6 hours was associated with greater post-procedural troponin T release and lower-threshold biomarker-defined myocardial injury, but not with 30-day MACE or a severe troponin T elevation of ≥70× URL. Therefore, our findings support a pharmacodynamically plausible biomarker association although the clinical significance of this association remains unclear.

In the present study, troponin T levels were measured using a conventional assay. Therefore, assay-specific differences in the interpretation of post-PCI troponin elevation should be considered. Previous studies have suggested that isolated post-procedural troponin elevation after PCI serves as a marker of adverse prognosis. Feldman et al. [17,18] reported that isolated post-PCI cardiac troponin I elevation after non-emergent PCI was associated with worse long-term survival, and their subsequent meta-analysis showed that post-procedural cardiac troponin I or T elevation was associated with increased long-term all-cause mortality and the composite of death and myocardial infarction. Testa et al. [19] also reported that troponin-defined PCI-related myocardial infarction according to the universal definition was associated with adverse in-hospital and 18-month outcomes. Subsequent studies attempted to identify clinically meaningful thresholds for post-PCI troponin elevation. Ferreira et al. [10] reported that troponin I elevation after elective PCI was associated with progressively higher 1-year mortality, with ≥5× URL appearing to be a stronger prognostic threshold. Silvain et al. [12] further refined this issue in a pooled patient-level analysis of patients who underwent elective PCI. Although ≥5× URL was associated with 1-year mortality in the overall population, their assay-specific interval analysis showed that, among patients assessed with conventional troponin, only ≥70× URL was significantly associated with 1-year mortality, whereas lower thresholds were more informative in patients assessed with high-sensitivity troponin. Herrmann et al. [11] reported that a conventional cardiac troponin T threshold of 25× the upper limit of normal provided optimal prognostic discrimination for 3-month mortality after PCI. Based on these prior data, we evaluated multiple thresholds, including ≥5× URL as a lower-threshold biomarker-defined injury endpoint, ≥25× URL as an intermediate threshold, and ≥70× URL as a severe injury threshold [10-13]. In our study, shorter clopidogrel preloading time was associated with troponin T elevations of ≥5× and ≥25× URL, but not with those of ≥70× URL or with 30-day MACE. Therefore, the observed association appears to reflect a lower-threshold biomarker release rather than severe prognostically established myocardial injury. However, the clinical significance of this finding remains unclear.

This study has several limitations. First, this was a single-center retrospective observational study, and causal relationships could not be established. Although stabilized IPTW was used to reduce the baseline imbalance between the clopidogrel preloading time groups, residual confounding is possible. In particular, the clopidogrel preloading time was not randomized but was determined by real-world clinical workflow, including admission pathways, catheterization laboratory scheduling, operator decision-making, and patient-related factors. Second, the 30-day clinical events were infrequent. The primary endpoint, 30-day MACE, occurred in only five patients, limiting the statistical power to detect differences in clinical outcomes between the groups. Therefore, the absence of a significant difference in 30-day MACE should not be interpreted as definitive evidence of the clinical equivalence between shorter and longer clopidogrel preloading times. Third, troponin T levels were measured using a conventional rather than a high-sensitivity assay. Conventional troponin assays have lower sensitivity than high-sensitivity assays for detecting small amounts of procedural myocardial injury, and the prognostic thresholds for post-PCI troponin elevation may differ according to the assay type [12]. Although we evaluated literature-based thresholds, including ≥5×, ≥25×, and ≥70× the URL, these biomarker analyses should be interpreted as exploratory. In particular, the lower-threshold troponin elevation observed in the present study may not necessarily represent clinically meaningful myocardial injury. Fourth, biomarker-defined periprocedural myocardial injury was primarily assessed based on post-procedural troponin T elevation. This should be distinguished from clinically defined periprocedural myocardial infarction, which requires additional ischemic evidence such as ischemic symptoms, electrocardiographic changes, imaging findings, and angiographic complications. Although we excluded patients with major procedural complications, including no-reflow, major coronary dissection, side branch occlusion, and distal embolization, minor procedural events or unmeasured lesion-related factors may have contributed to post-procedural troponin release. Fifth, although patients with unstable angina and myocardial infarction were excluded, the retrospective nature of this study limited our ability to fully assess pre-procedural symptoms, ischemic burden, and clinical stability. Therefore, unmeasured differences in patient presentation or lesion vulnerability may have influenced both clopidogrel preloading time and post-procedural biomarker release. Sixth, this study specifically included patients who were clopidogrel-naive and received 300 mg clopidogrel preloading within 24 hours before PCI. Therefore, the findings may not be generalizable to patients receiving 600 mg clopidogrel loading or more potent P2Y12 inhibitors, which have a faster onset of platelet inhibition and are increasingly used in selected patients. Nevertheless, focusing on a homogeneous 300 mg clopidogrel preloading population reduced treatment heterogeneity and allowed a more direct evaluation of preloading-time effects. Finally, the study was conducted in a Korean population treated with second-generation drug-eluting stents. Therefore, caution is required when extrapolating these findings to non-Korean populations, patients with different genetic or metabolic backgrounds, and patients treated using other stent platforms or contemporary PCI strategies. Further prospective studies with standardized clopidogrel dosing, systematic assessment of ischemic criteria, high-sensitivity troponin measurements, and longer-term follow-up are needed to clarify the clinical significance of post-procedural troponin elevation according to clopidogrel preloading times.

In conclusion, clopidogrel preloading ≤6 hours before elective PCI was not associated with increased 30-day MACE but was associated with greater post-procedural troponin T release and lower-threshold troponin T elevation. This association was not observed for severe troponin T elevation of ≥70× URL, and the clinical significance of these biomarker findings requires further investigation.

## Figures and Tables

**Table 1.** Baseline characteristics according to clopidogrel preloading time

Characteristic	Before IPTW			After IPTW		
	Preloading ≤6 hr (n=260)	Preloading >6 hr (n=760)	SMD	Preloading ≤6 hr	Preloading > 6 hr	SMD
Age (yr)	65.8±11.5	65.3±9.8	0.034	65.4±11.2	65.3±9.8	0.009
Male sex	174 (66.9)	509 (67.0)	0.000	69.0	66.8	0.047
Hypertension	174 (66.9)	512 (67.4)	0.020	66.8	66.4	0.009
Diabetes mellitus	100 (38.5)	322 (42.4)	0.081	40.1	41.2	0.021
Current smoker	61 (23.5)	175 (23.0)	0.010	22.6	23.2	0.015
Previous myocardial infarction	24 (9.2)	39 (5.1)	0.145	5.6	5.4	0.008
Previous stroke	24 (9.2)	81 (10.7)	0.005	8.6	10.0	0.049
Hemoglobin (g/dL)	13.4±2.1	13.7±3.6	0.110	13.6±2.1	13.7±3.3	0.017
eGFR (mL/min/1.73 m²)	78.5±28.2	76.3±24.2	0.102	77.3±26.0	77.4±24.2	0.002
Baseline CK-MB (ng/mL)	3.51±6.54	3.08±12.89	0.050	3.71±7.73	3.06±13.35	0.060
Baseline troponin T (ng/mL)	0.009±0.005	0.009±0.004	0.111	0.009±0.005	0.009±0.004	0.002
LDL cholesterol (mg/dL)	99.3±37.2	105.0±39.9	0.150	102.6±38.6	103.9±39.4	0.033
Culprit lesion						
Left main	16 (6.2)	65 (8.6)	0.094	6.4	9.2	0.104
Left anterior descending	173 (66.5)	484 (63.7)	0.057	65.1	63.5	0.033
Left circumflex	38 (14.6)	102 (13.4)	0.031	14.2	13.7	0.012
Right coronary artery	33 (12.7)	109 (14.3)	0.040	14.3	13.5	0.023
Multivessel disease	122 (46.9)	330 (43.4)	0.077	46.1	44.9	0.024
Chronic total occlusion	22 (8.5)	64 (8.4)	0.010	8.9	8.2	0.025
Stent overlap	31 (11.9)	94 (12.4)	0.017	12.7	12.0	0.022
Two stents in bifurcation lesion	6 (2.3)	14 (1.8)	0.037	2.2	1.8	0.026
Stent number per patient	1.52±0.84	1.62±0.95	0.079	1.57±0.82	1.64±0.96	0.074
Minimal stent diameter (mm)	2.92±0.44	2.90±0.39	0.025	2.93±0.44	2.90±0.39	0.058
Total stent length (mm)	42.2±27.5	42.9±28.1	0.031	42.4±28.2	42.9±28.1	0.018
Maximal balloon pressure (atm)	14.2±3.1	14.8±4.5	0.155	14.6±3.1	14.6±4.2	0.015
Discharge medications						
Aspirin	259 (99.6)	760 (100.0)	0.091	99.8	100.0	0.056
Clopidogrel	227 (87.3)	693 (91.2)	0.081	90.2	90.4	0.007
Beta blocker	121 (46.5)	466 (61.3)	0.339	56.0	57.2	0.025
ACEI/ARB	203 (78.1)	664 (87.4)	0.270	84.9	84.8	0.003
Statin	254 (97.7)	755 (99.3)	0.143	99.0	99.1	0.006

Values are presented as mean±standard deviation, number (%), weighted mean±standard deviation, or weighted percentages, as appropriate. Absolute counts represent the original sample size. Standardized mean differences (SMD) were calculated to assess covariate balance, with an absolute value <0.10 indicating adequate balance. Weighted estimates were derived using inverse probability of treatment weighting (IPTW). eGFR, estimated glomerular filtration rate; CK-MB, creatine kinase MB fraction; LDL, low-density lipoprotein; ACEI, angiotensin-converting enzyme inhibitor; ARB, angiotensin receptor blocker.

**Table 2.** Thirty-day clinical outcomes according to clopidogrel preloading time using the 6-hour cutoff

Outcome	Before IPTW				After IPTW			
	Preloading ≤6 hr (n=260)	Preloading >6 hr (n=760)	OR (95% CI)	*p*-value	Preloading ≤6 hr	Preloading > 6 hr	OR (95% CI)	*p*-value
Ischemic events								
All-cause death	1 (0.4)	0 (0)	8.79 (0.36–216.50)	0.255	0.2	0.0	5.29 (0.16–171.03)	0.347
Myocardial infarction	1 (0.4)	2 (0.3)	1.46 (0.13–16.21)	>0.999	0.4	0.3	1.47 (0.12–18.22)	0.762
Stroke	0 (0)	1 (0.1)	0.97 (0.04–23.93)	>0.999	0.0	0.1	1.06 (0.04–26.75)	0.971
Stent thrombosis	1 (0.4)	2 (0.3)	1.46 (0.13–16.21)	>0.999	0.4	0.3	1.47 (0.12–18.22)	0.762
Any revascularization	1 (0.4)	2 (0.3)	1.46 (0.13–16.21)	>0.999	0.4	0.3	1.47 (0.12–18.22)	0.762
MACE^a)^	2 (0.8)	3 (0.4)	1.96 (0.33–11.77)	0.606	0.5	0.4	1.40 (0.17–11.38)	0.754
Bleeding events								
BARC 3A	2 (0.8)	2 (0.3)	2.94 (0.41–20.96)	0.270	0.1	0.2	0.57 (0.01–22.35)	0.761
BARC 3B	0 (0)	4 (0.5)	0.32 (0.02–6.01)	0.577	0.0	0.5	0.36 (0.02–6.73)	0.492
BARC 3C	1 (0.4)	0 (0)	8.79 (0.36–216.50)	0.255	0.7	0.0	12.83 (0.59–280.64)	0.105
BARC 5	0 (0)	0 (0)			0.0	0.0		
BARC 3 or 5	3 (1.2)	6 (0.8)	1.47 (0.36–5.91)	0.701	0.8	0.8	1.07 (0.21–5.53)	0.932
Net events (%)	5 (1.9)	9 (1.2)	1.64 (0.54–4.93)	0.377	1.3	1.1	1.18 (0.33–4.32)	0.797

Values are presented as number (%) or weighted percentages. Odds ratios (ORs) and 95% confidence intervals (CIs) compare patients with clopidogrel preloading ≤6 hours with those with clopidogrel preloading >6 hours in the crude and inverse probability of treatment weighting (IPTW) analyses. Because several rare outcomes had zero cells, 0.5 continuity correction was applied for OR and 95% CI estimation where appropriate. BARC, Bleeding Academic Research Consortium.

a) Major adverse cardiovascular events (MACE) indicate composite of all-cause death, myocardial infarction, stroke, and any revascularization.

**Table 3.** Serial changes in troponin T according to clopidogrel preloading time using the 6-hour cutoff

Time point	Preloading ≤6 hr			Preloading > 6 hr			*p*-value
	Number	Geometric mean (95% CI)	Median (IQR)	Number	Geometric mean (95% CI)	Median (IQR)	
Baseline	260	0.0075 (0.0068–0.0082)	0.0100 (0.0060–0.0140)	760	0.0076 (0.0073–0.0079)	0.0090 (0.0050–0.0140)	0.847
6 hr after PCI	257	0.0462 (0.0386–0.0554)	0.0310 (0.0150–0.1180)	754	0.0290 (0.0263–0.0319)	0.0240 (0.0110–0.0570)	<0.001
24 hr after PCI	260	0.0592 (0.0497–0.0706)	0.0510 (0.0187–0.1693)	758	0.0433 (0.0392–0.0479)	0.0340 (0.0160–0.1030)	<0.001
48 hr after PCI	238	0.0572 (0.0471–0.0693)	0.0475 (0.0170–0.1440)	719	0.0396 (0.0357–0.0439)	0.0320 (0.0150–0.0930)	<0.001

Troponin T values (ng/mL) are expressed as geometric mean (95% confidence interval [CI]) and median (interquartile range [IQR]). Because troponin T values were right-skewed, values were log-transformed after adding a small offset. Serial changes were compared using a linear mixed-effects model with a random intercept for patients. PCI, percutaneous coronary intervention.

**Table 4.** Peak troponin T according to cumulative clopidogrel preloading time cutoffs

Comparison (hr)	≤Cutoff			>Cutoff			Geometric mean ratio^a)^ (95% CI)	*p*-value	
	Number	Geometric mean (95% CI)	Median (IQR)		Geometric mean (95% CI)	Median (IQR)		Log-scale *t*-test	Mann-Whitney U-test
≤1 vs. >1	91	0.103 (0.075–0.142)	0.100 (0.028–0.364)	929	0.048 (0.044–0.052)	0.036 (0.017–0.110)	2.12 (1.53–2.95)	<0.001	<0.001
≤2 vs. >2	126	0.087 (0.065–0.115)	0.084 (0.025–0.272)	894	0.047 (0.043–0.052)	0.036 (0.017–0.109)	1.80 (1.35–2.40)	<0.001	<0.001
≤4 vs. >4	230	0.067 (0.055–0.081)	0.057 (0.019–0.173)	790	0.047 (0.043–0.052)	0.035 (0.017–0.111)	1.40 (1.13–1.73)	0.002	0.003
≤6 vs. >6	260	0.064 (0.054–0.077)	0.051 (0.019–0.176)	760	0.047 (0.043–0.052)	0.036 (0.017–0.109)	1.36 (1.11–1.66)	0.003	0.004
≤8 vs. >8	406	0.060 (0.052–0.068)	0.045 (0.019–0.146)	614	0.046 (0.041–0.052)	0.035 (0.016–0.110)	1.28 (1.07–1.52)	0.006	0.005
≤12 vs. >12	496	0.056 (0.050–0.064)	0.041 (0.019–0.137)	524	0.047 (0.041–0.053)	0.036 (0.016–0.110)	1.21 (1.02–1.43)	0.033	0.052

Troponin T values (ng/mL) are expressed as geometric mean (95% confidence intervals [CIs]) and median (interquartile range [IQR]). Because troponin T values were right-skewed, the primary comparison used log-transformed peak troponin T; Mann-Whitney U-test results are also shown as a nonparametric sensitivity comparison.

a) Ratio of geometric means in the ≤cutoff group relative to the >cutoff group.

**Table 5.** Biomarker outcomes according to clopidogrel preloading time using the 6-hour cutoff

Biomarker endpoint	Before IPTW				After IPTW			
	Preloading ≤6 hr (n=260)	Preloading > 6 hr (n=760)	OR (95% CI)	*p*-value	Preloading ≤6 hr	Preloading >6 hr	OR (95% CI)	*p*-value
TnT elevation								
≥5× URL	112 (43.1)	255 (33.6)	1.50 (1.12–2.00)	0.006	43.6	33.7	1.52 (1.13–2.04)	0.006
≥25× URL	43 (16.5)	66 (8.7)	2.08 (1.38–3.15)	<0.001	15.4	8.3	2.03 (1.31–3.14)	0.002
≥70× URL	8 (3.1)	26 (3.4)	0.90 (0.40–2.01)	0.790	3.2	3.5	0.90 (0.40–2.05)	0.807

Values are number (%) or weighted percentages. IPTW, inverse probability of treatment weighting; OR, odds ratio; CI, confidence interval; TnT, troponin T; URL, upper reference limit.

**Table 6.** Multivariable logistic regression analysis for biomarker-defined periprocedural myocardial injury

Predictor	TnT elevation ≥5× URL		TnT elevation ≥25× URL		TnT elevation ≥70× URL		
	OR (95% CI)	*p*-value	OR (95% CI)	*p*-value	OR (95% CI)	*p*-value	
Clopidogrel preloading ≤6 hr	1.50 (1.10–2.05)	0.010	2.15 (1.38–3.34)	<0.001	0.83 (0.36–1.90)	0.658	
Age >65 yr	1.23 (0.92–1.65)	0.156	0.99 (0.63–1.57)	0.978	0.51 (0.24–1.07)	0.076	
Male sex	0.93 (0.69–1.27)	0.665	1.46 (0.90–2.37)	0.130	1.41 (0.63–3.15)	0.407	
Diabetes mellitus	0.88 (0.66–1.17)	0.375	1.11 (0.71–1.73)	0.645	1.63 (0.77–3.42)	0.200	
Multivessel disease	1.97 (1.45–2.67)	<0.001	1.91 (1.19–3.06)	0.007	2.07 (0.94–4.54)	0.069	
Stent length >33 mm	1.32 (0.98–1.79)	0.071	1.22 (0.76–1.94)	0.413	0.96 (0.44–2.07)	0.913	
Hemoglobin <12 g/dL	2.26 (1.54–3.32)	<0.001	2.87 (1.73–4.79)	<0.001	2.10 (0.89–4.93)	0.089	
eGFR <60 mL/min/1.73 m²	2.89 (2.04–4.11)	<0.001	3.51 (2.20–5.61)	<0.001	3.34 (1.51–7.41)	0.003	

TnT, troponin T; URL, upper reference limit; OR, odds ratio; CI, confidence interval; eGFR, estimated glomerular filtration rate.
